# Determining spatio-temporal distribution and hotspots for female schistosomiasis in Ghana, 2020–2024: a call for increased public health action against a neglected tropical disease

**DOI:** 10.1186/s40249-026-01452-7

**Published:** 2026-05-13

**Authors:** Ebenezer Efichie, Christabel Gyebuaa Mensah, Jochoniah Muatha Nzomo

**Affiliations:** 1 Ghana Health Service, Kwahu Afram Plains North District Health Directorate, Donkorkrom, Ghana; 2 Department of Epidemiology and Biostatistics, Kwame Nkrumah University of Science and Technology, Kumasi, Ghana

**Keywords:** Female schistosomiasis, Spatiotemporal, Ghana, Neglected tropical diseases

## Abstract

**Background:**

Schistosomiasis remains a major neglected tropical disease in Ghana with substantial health and social consequences for women and girls. Long-standing mass drug administration (MDA) programmes with praziquantel have been implemented since 2008, yet the disease remains endemic in Ghana raising significant concerns about World Health Organization (WHO) efforts towards elimination by 2030. The study thus aimed to assess the spatio-temporal and hotspots for female schistosomiasis (FS) in Ghana and provide insight that informs gender-sensitive public health interventions.

**Methods:**

We conducted an ecological, retrospective, spatio-temporal analysis using routine surveillance data on FS. Case counts from female population data were extracted from the District Health Information Management System 2 (DHIMS-2) for all 260 districts in Ghana between 2020 and 2024. Annual and period prevalence per 10,000 female populations were estimated. Global Moran’s *I* was used to assess spatial autocorrelation, and clustering and hotspots were assessed using Anselin Local Moran’s I and Getis-Ord Gi* statistics respectively, with False Discovery Rate (FDR) correction.

**Results:**

A total of 4864 FS cases were reported over the five years, with a significant increase in prevalence from 71.9 cases per 10,000 female population in 2020 to 99 cases per 10,000 female population in 2024. Majority of the cases occurred in the second half of the year. There was an observed diffusion of prevalence of the cases from the east-southern to west-southern part of the country. Spatial autocorrelation was observed for FS (Moran I index = 0.060032, Z-Score = 2.650757 and a *P* < 0.008). Ten districts showed high-high clustering, with districts in middle and south-western zones of the country showing significant hotspots for female schistosomiasis in Ghana.

**Conclusions:**

The occurrence of female schistosomiasis is not a coincidence. Female populations in approximately 6.5% hotspot districts are at significant risk of female schistosomiasis infection. Hence, gender sensitive schistosomiasis control, including targeted MDA, should be directed towards clustered hotspots, focusing on zones with low prevalence fenced off by high prevalence zones.

**Graphical Abstract:**

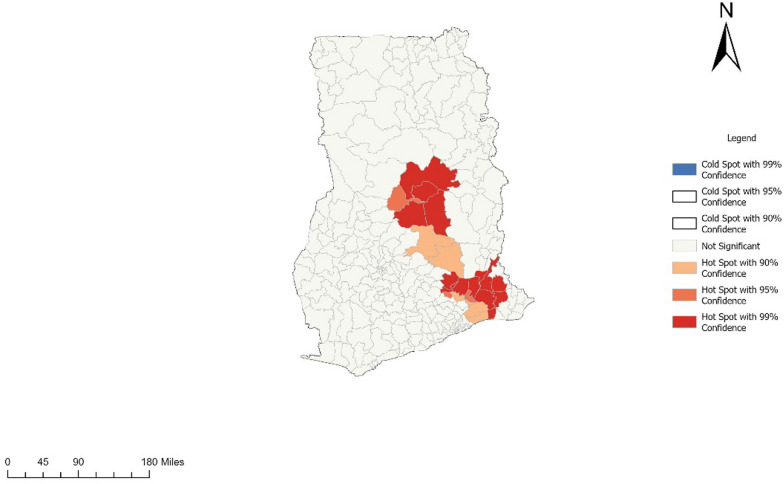

## Background

Schistosomiasis is an infectious parasitic disease caused by parasitic blood flukes of the genus *Schistosoma*. It is often termed “*a disease of poverty*”, and is acquired through penetration of the skin by the blood fluke when people come into contact with freshwater infested with larval form (cercariae) [1], which penetrate the skin and can lead to chronic health problems and social stigma [2]. Globally, about 251.4 million people need treatment for schistosomiasis with about 90% of these cases concentrated in Africa [3].

Recognizing the persistent and disproportionate burden of schistosomiasis in low-income settings, the World Health Organization (WHO) 2021–2030 Roadmap for Neglected Tropical Diseases set an ambitious target of eliminating schistosomiasis as a public health problem in all endemic countries by 2030, with a global goal of achieving a 90% reduction in the number of people requiring interventions against neglected tropical diseases (NTDs) by the same year [4]. To operationalize these targets, updated WHO guidelines published in 2022 established a single 10% prevalence threshold of *Schistosoma* spp. to initiate preventive chemotherapy, applicable to all at-risk population groups, replacing earlier frameworks that primarily targeted school-aged children (SAC) [5]. Complementing these treatment guidelines, a WHO monitoring and evaluation (M&E) framework for schistosomiasis and soil-transmitted helminthiases was developed to guide countries in assessing programme progress, adjusting treatment frequency from twice-yearly in high-burden settings to annual, biennial, and periodic treatment as prevalence declines, and tracking progress toward eliminating schistosomiasis as a public health problem at national and sub-national levels and reaching the thresholds [6]. Understanding the spatial distribution of female schistosomiasis at the district level is, therefore, directly relevant to these global and national M&E imperatives.

Within this overall burden, female schistosomiasis, which includes both urogenital and intestinal forms in women and girls, leads to chronic health complications such as anaemia, infertility and increased vulnerability to other infections [2]. However, regardless of these serious consequences, many aspects of female schistosomiasis remain neglected in research, surveillance, and control programs, even though four out of every ten schistosomiasis cases were reported among females in 2024 [3, 7].

It is important to distinguish female schistosomiasis broadly from female genital schistosomiasis (FGS), a specific and particularly debilitating manifestation caused predominantly by *Schistosoma haematobium*, in which parasite eggs become trapped in the tissues of the female genital tract, leading to gynaecological and reproductive pathology. FGS is estimated to affect up to 56 million women and girls across sub-Saharan Africa, yet it remains profoundly under-diagnosed and under-treated; despite this massive at-risk population, fewer than 15,000 women have been formally screened for FGS to date [8, 9]. Population-based studies across sub-Saharan Africa report FGS prevalence ranging from 10.6% to 75% in *Schistosoma*-endemic areas, with *S. haematobium* being the predominant causative species. In Ghana specifically, a study conducted in the Volta Basin reported FGS prevalence as high as 79.5%, predominantly among the 11–20 age group, underscoring the severity of the disease burden in Ghanaian women and girls [10]. Beyond reproductive morbidity, FGS is further associated with increased risk of human immunodeficiency virus (HIV) acquisition, high-risk human papillomavirus (HPV), *Trichomonas vaginalis* infection, and cervical precancerous lesions, compounding the public health relevance of this neglected condition for women's health systems in Ghana and across the continent.

Ghana's response to schistosomiasis has been one of the more systematically documented in West Africa. The Ghana Health Service launched its National Neglected Tropical Disease Programme in 2008, building on baseline disease mapping that was first initiated in 2007 and progressively expanded to cover additional districts. To date, 202 districts across 554 sub-districts have been surveyed for schistosomiasis, and all 261 districts have been treated with praziquantel mass drug administration (MDA) [11]. These sustained programmatic efforts have yielded measurable gains: overall schistosomiasis prevalence declined dramatically from 21.1% at baseline (2007–2010) to 3.5% at the first impact assessment in 2015, and further to 6.8% by the most recent impact assessment in 2024 [11]. This decrease has been largely attributed to consistent therapeutic pressure through treating whole communities in high-prevalence areas (> 50%) and treating all school-aged children in areas of moderate and low endemicity. Notwithstanding these achievements, challenges persist: logistical constraints, variable treatment coverage, reinfection from untreated water sources, and the near-exclusive focus of MDA programmes on school-aged children has left adult community members, particularly women engaged in water-contact activities, without adequate treatment coverage [12]. The persistence of endemic transmission in the Volta Lake basin districts and parts of southern Ghana, even after fifteen years of MDA, points to entrenched environmental and socio-behavioural determinants of exposure that pharmacological interventions alone cannot resolve.

Because schistosomiasis transmission is strongly shaped by place and time, understanding the spatial and temporal dynamics of disease transmission is crucial for clarifying the epidemiology of female schistosomiasis. Previous studies have demonstrated that environmental factors such as proximity to water bodies and climatic conditions play an essential role in disease distribution [13, 14]. However, in Ghana, limited research has focused specifically on the spatial burden of female schistosomiasis among women, with most studies either not using spatial or focusing on school children in the general population [15, 16]. Consequently, important questions remain about how the burden of female schistosomiasis varies across districts and over time, despite ongoing control efforts.

It was against this background that this study was conducted to bridge this gap by mapping the spatiotemporal distribution and hotspots of female schistosomiasis across Ghana over a five-year period. By identifying districts with consistently high or emerging burden among females, the study aims to provide evidence that can inform gender-sensitive public health interventions in identified hotspot areas.

## Methods

### Study design

We conducted an ecological, retrospective, spatio-temporal analysis of routine surveillance data on female schistosomiasis in Ghana. The analysis used district-level female schistosomiasis case counts and corresponding female population estimates reported through the District Health Information Management System 2 (DHIMS-2) for the period 2020–2024.

### Study setting

The study was conducted in Ghana among all Metropolitans, Municipals, and Districts (MMDAs). The country lies on the West Coast of Africa between Latitudes 5^o^ and 11^o^ North of the equator and between longitude 1^o^ East and 3^o^ West of the zero meridian. The combination of low altitude and proximity to the equator gives Ghana a typical tropical climate [17].

**Fig. 1:**
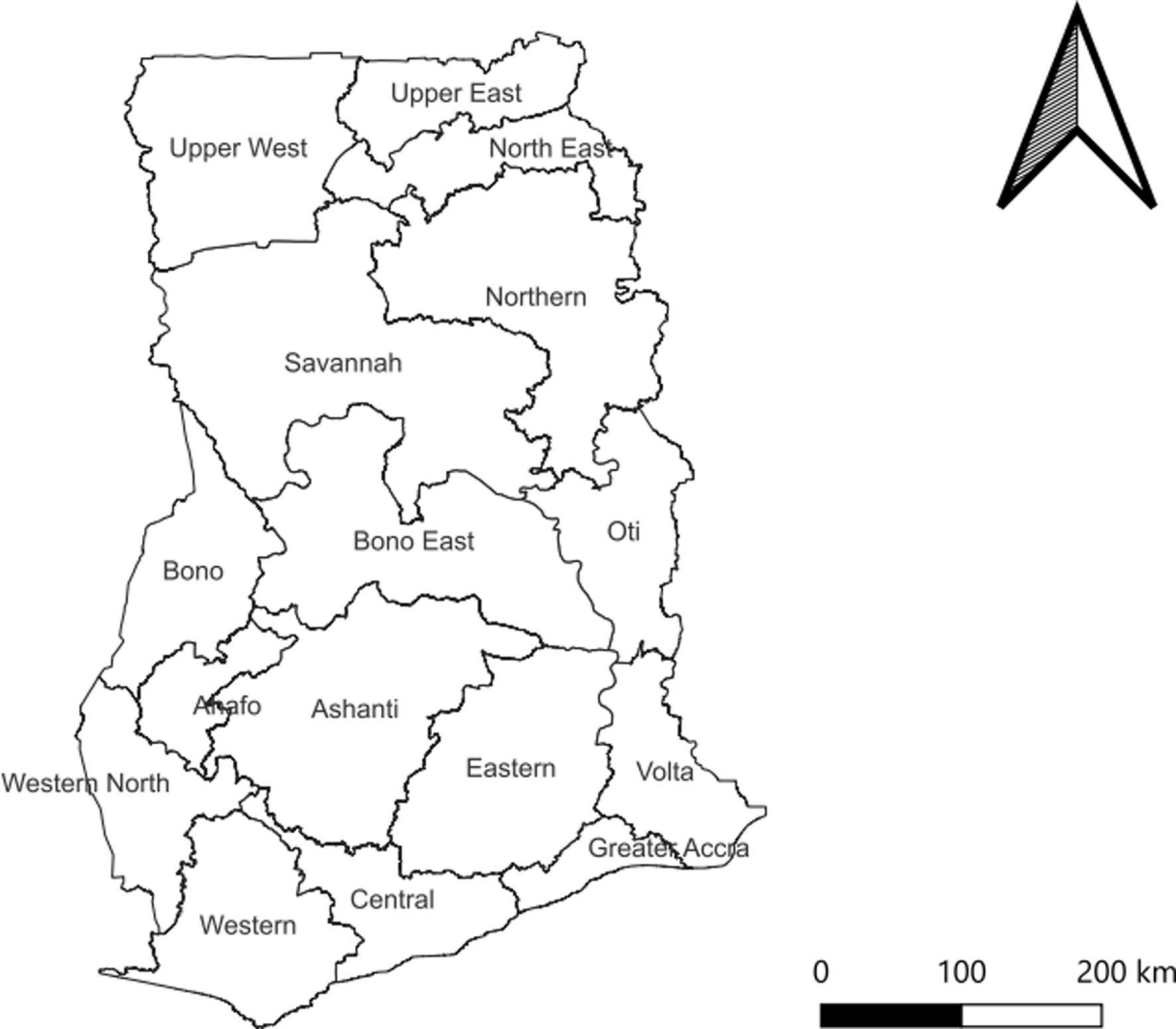
Base map of study area in Ghana

**Fig. 2:**
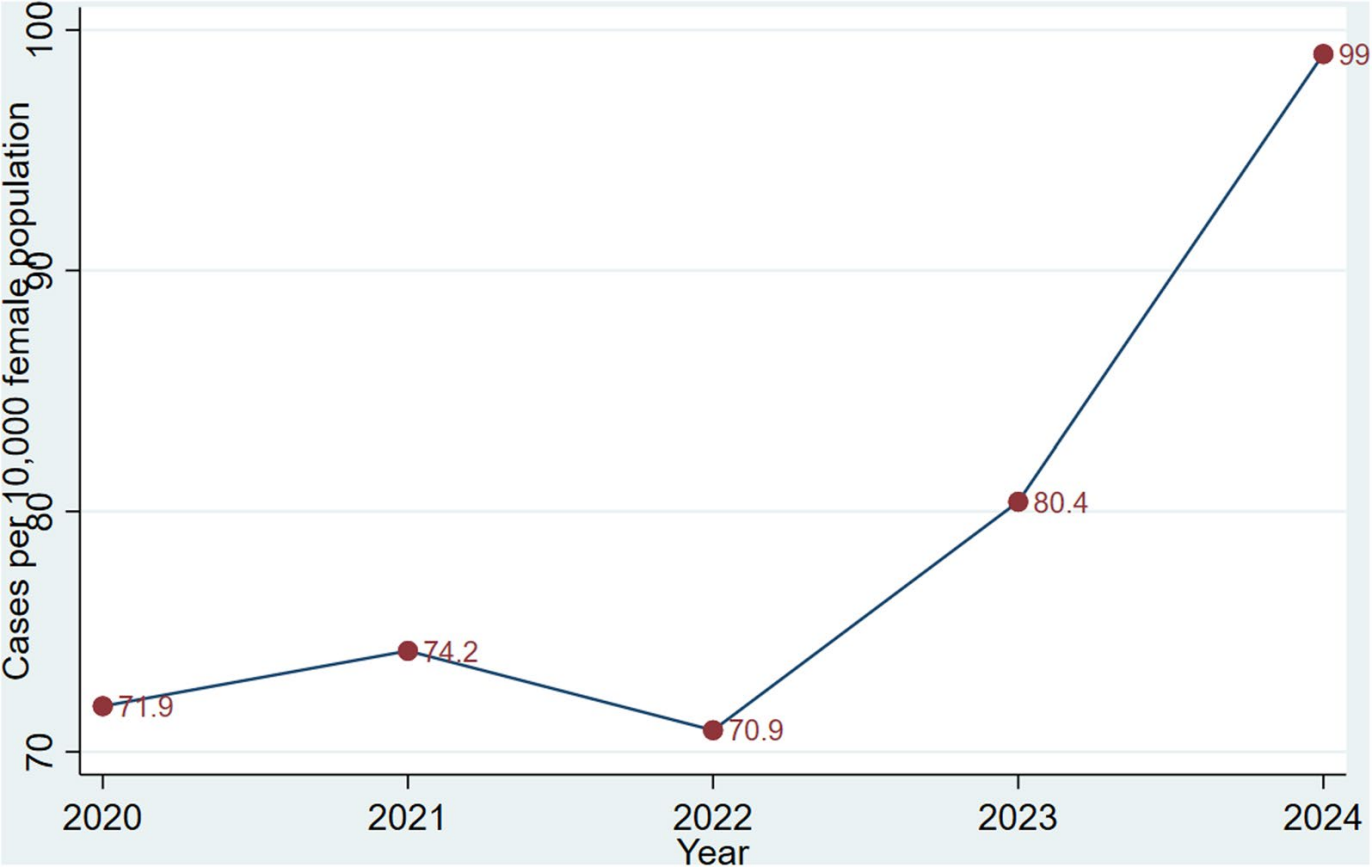
Temporal prevalence in female schistosomiasis in Ghana during 2020–2024

**Fig. 3:**
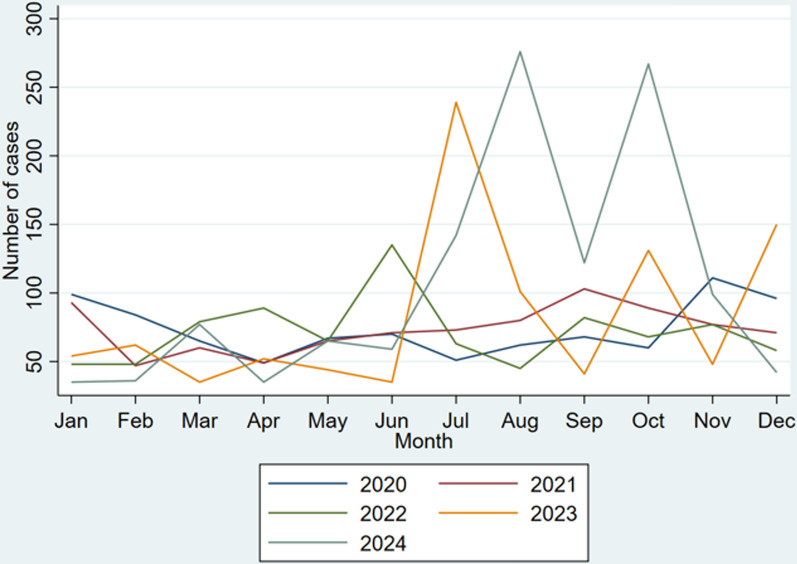
Monthly trend in female schistosomiasis cases in Ghana during 2020–2024

**Fig. 4:**
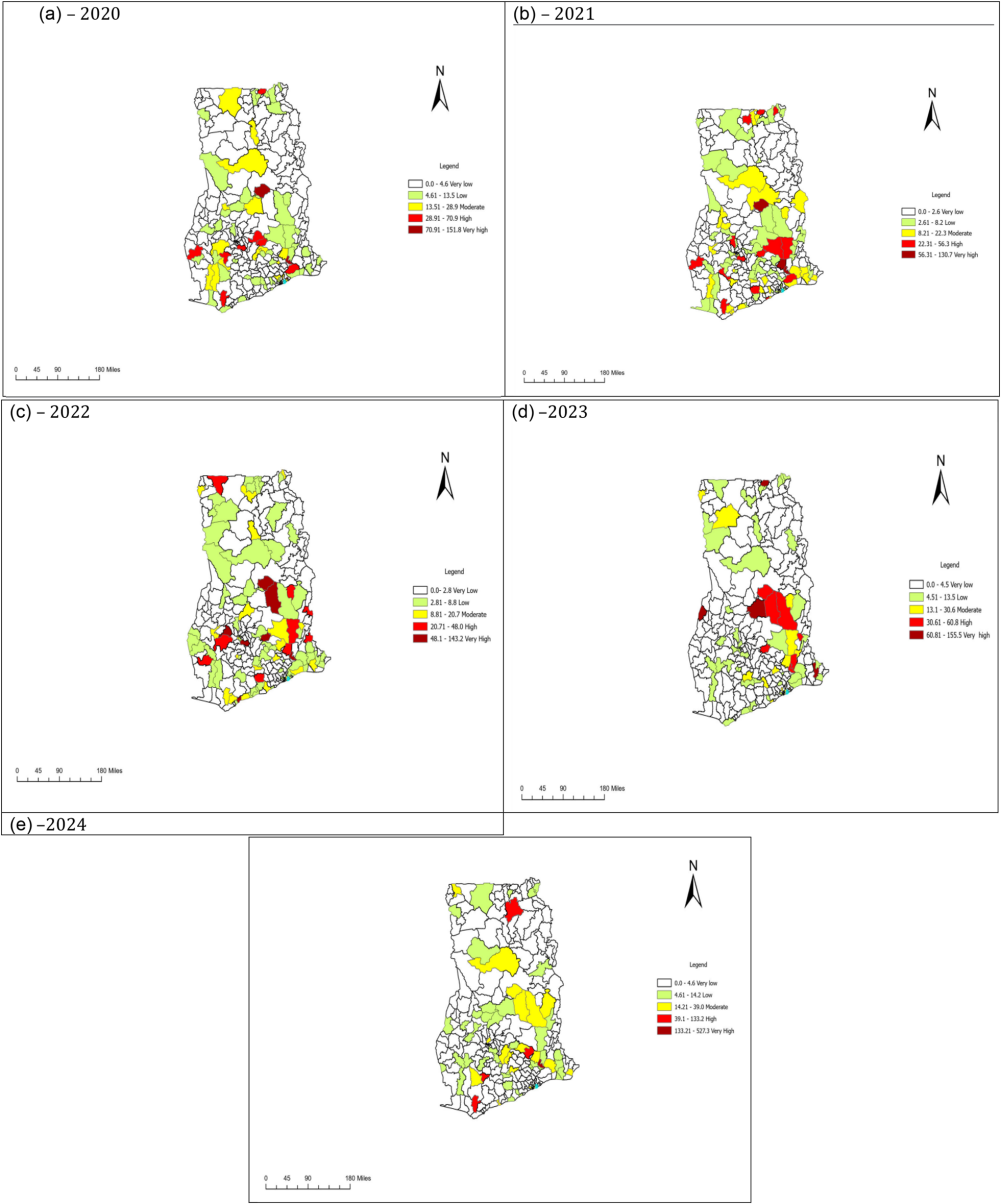
(**a**–**e**) Spatio-temporal distribution of female schistosomiasis by districts in Ghana, 2020–2024

**Fig. 5:**
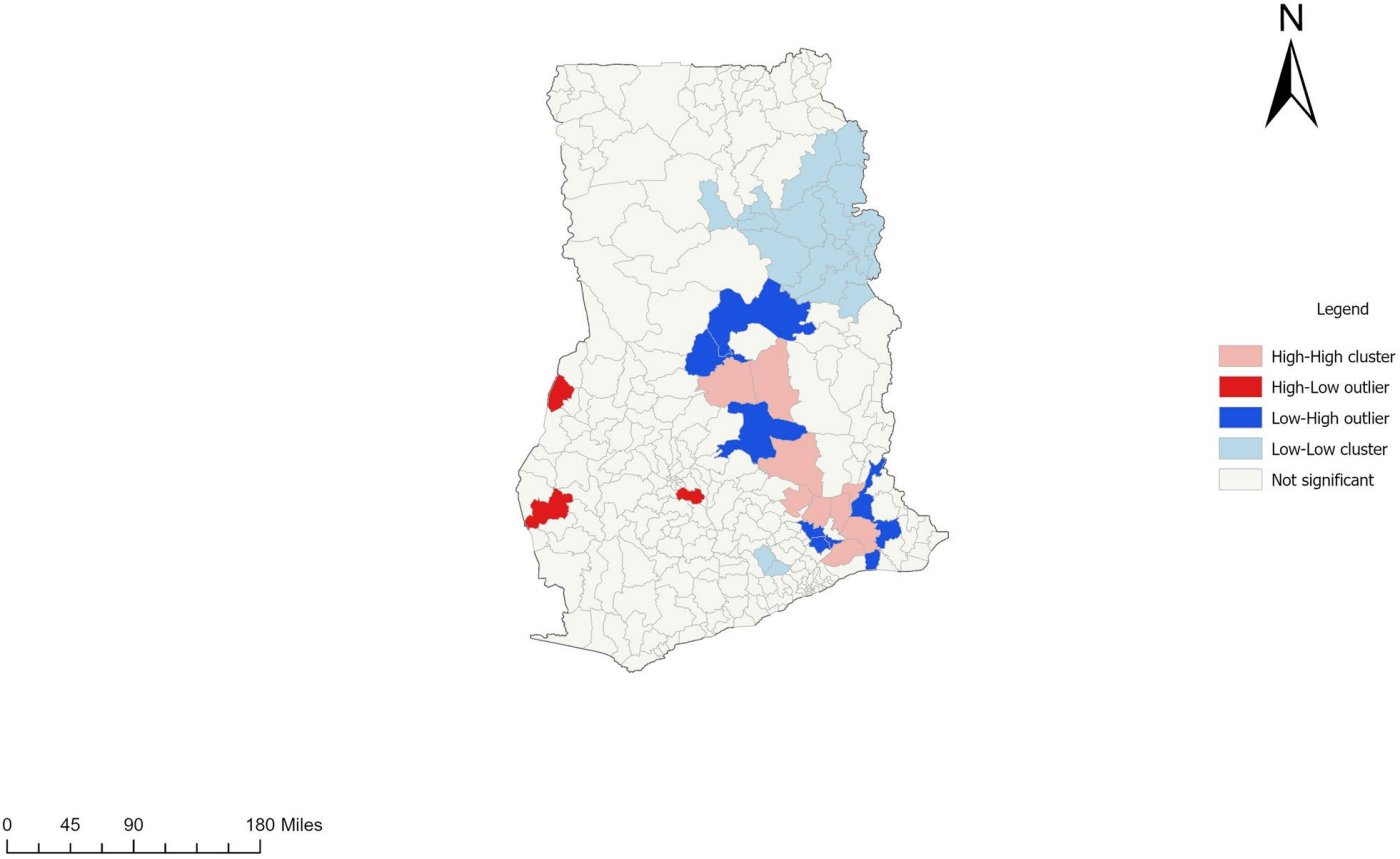
Local Indicators of Spatial Association result showing local spatial autocorrelation in districts in Ghana, 2020–2024

The rainfall figures are highest in the forested southwest and lowest in the north. The rainy season is different for the norther and southern part of the country with the southern part having two rainy seasons in the year, and the northern having one rain season. Out of the 30.8 million population of the country, 50.7% are females. About 43.3% are rural residents, with the majority of them engaging in agriculture as their main economic activity [17] (Figs. 1, 2, 3, 4, 5).

### Data collection

Data were extracted from DHIMS-2, a routine health information database of the Ghana Health Service, in an Excel format for the period 2020 to 2024. The data were extracted based on 260 district-level organization units and were checked to include only female schistosomiasis cases for the selected period. The number of cases in the neglected tropical disease (NTD) dataset was cross-checked with cases reported in the monthly Integrated Disease Surveillance and Response (IDSR) dataset to ensure consistency and accuracy of cases reported. The annual female population for 2020 to 2024 were extracted from DHIMS-2. Female population values with decimals were rounded to the nearest whole number. The annual female population of all the 260 districts was extracted and used to estimate the prevalence of female schistosomiasis in each district. The Guan district was excluded from the current 261 districts in Ghana due to its recent creation and the unavailability of data in the shapefile.

### Data processing and analysis

To depict the temporal trend in female schistosomiasis cases, the data were structured according to months, year and cases in Microsoft Excel 2016. The extracted female schistosomiasis and population data were matched to their corresponding districts on the shapefile in ArcGIS Pro 3.0 software (Esri, Redlands, CA, USA) [18]. The software was used due to its ability to map spatial data and determine areas with high prevalence of events. The data downloaded from DHIMS-2 and cleaned were merged using an Object Identifier (OID) in the data and a Feature Identifier (FID) in the shapefile respectively.

### Descriptive analysis

Female schistosomiasis data were aggregated across 2020–2024 and normalized for population by the estimated population. For each year, annual prevalence was calculated as the number of reported female schistosomiasis cases per 10,000 female population in each district. Line graphs were used to depict the annual and monthly temporal trends in cases using STATA 17 (StataCorp LLC, College Station, Texas, USA) [19] and choropleth maps were created to depict the spatial distribution of female schistosomiasis.

### Assessing spatial autocorrelation of female schistosomiasis cases in Ghana

Spatial autocorrelation was assessed using Moran’s *I* statistics, both global and local. The spatial units were examined to determine where female schistosomiasis clustered in regions in Ghana. This was to generate a risk map for female schistosomiasis in areas that are narrowed to the district level to aid in developing a targeted response. Additionally, this analytical instrument is sensitive to eliciting critical details for priority setting and decision-making.

The Global Moran’s *I* statistic provides a measure of the spatial autocorrelation (tendency of neighbouring regions to have similar values), where a positive value indicates positive spatial autocorrelation, and a negative value indicates negative spatial autocorrelation [20].

Local spatial autocorrelation was examined using Anselin Local Moran’s *I,* which compares the similarity of risk between a district and its neighbours. A positive value indicates similar risk, while a negative value indicates dissimilar risk [18]. This was used to obtain clusters and outliers. For example, a result of low–high represents a district with low risk surrounded by districts with high risk. The tools were run in ArcGIS pro 3.0 using polygon contiguity. For the spatial autocorrelation, Moran’s *I* statistic is given as:

$$\mathrm{I} = \frac{n}{{S}_{0}} \frac{\sum_{i=1}^{n}\sum_{j=1}^{n}{w}_{i.j}{z}_{i}{z}_{j}}{\sum_{i=1}^{n}{z}_{i}^{2}}$$

For feature i, zi is the attribute’s deviation from its mean (xi- X¯). As shown in the equation, wi.j is the spatial weight between features i and j, n represents the number of features, and S_0_ is the sum of all spatial weights:

$${S}_{0}={\sum}_{i=1}^{n}{\sum}_{j=1}^{n}{w}_{i.j}$$

The z_I_-score statistic is calculated as follows:

$${z}_{I}= \frac{I - E [I]}{\sqrt{V [I]}}$$

where

$${\mathrm{V}}\left[ {\mathrm{I}} \right] \, = {\mathrm{E}}\left[ {{\mathrm{I}}^{{2}} } \right]{-}{\mathrm{E}}\left[ {\mathrm{I}} \right]^{{2}}$$

### Analyzing female genital schistosomiasis hotspots

For each polygon representing a district in the dataset, the hotspot analysis tool was used to calculate the Getis-Ord Gi*statistic (Esri, Redlands, CA, USA) [18]. The generated z-scores and *P*-values show the locations of where high- and low-value features are spatially grouped. This tool analyses each feature in relation to its surroundings. A high-value feature is intriguing, but it may not be a statistically significant hotspot. In order to be a statistically significant hotspot, a feature must have a high value and be surrounded by other features with high values [18]. The study used False Discovery Rate (FDR) correction to adjust for multiple testing and spatial dependence.

The formula is given below:

$${G}_{i}^{*}= \frac{{\sum}_{i=1}^{n}{w}_{i.j}{x}_{j }- \overline{X } {\sum }_{i=1}^{n}{w}_{ij}}{S\sqrt{\frac{\left[n{\sum}_{i=1}^{n}{w}_{ij}^{2}-({\sum}_{i=1}^{n}{w}_{ij}{)}^{2}\right]}{n-1}}}$$

wj is the feature j attribute value, and wi*,*j takes into account the spatial weight between feature i and j*; n* is sum of features. As with the Global Moran *I* analysis, spatial relationships were conceptualized using polygon contiguousness with edges and corners. Incremental spatial auto-correlation analysis was conducted to choose a precise and realistic search radius that agrees with the neighbourhood conceptualization. The analysis was performed using the Euclidean Distance method. Following the outcome of the computation, the highest peak distance value (56,234.94 m) was chosen as the distance bandwidth or search radius.

### Ethical approval and consent to participate

Aggregate secondary data were used for the study. Therefore, no ethical approval was required. However, permission was obtained from the Ghana Health Service to use the data. The data used did not contain any personal identifiers.

## Results

### Descriptive epidemiology of female schistosomiasis

A total of 4864 cases of female schistosomiasis (FS) were reported from 2020–2024, with 2024 having the highest count number of cases (1255) and 2022 recording the least count cases (857). Further, 2024 recorded the highest burden of FS (99.0/10,000 female population) and 2022 recorded the least burden of FS (70.9/10,000 female population) period prevalence (See Fig. 2).

It was further observed that the incidence of FS followed a cyclical trend with most cases reported in the second half of each year (See Fig. 3).

### Spatial distribution of female schistosomiasis

From 2020 to 2024, there was an observed decline (changing from deep red to yellow or green) in FS burden among districts in the northern part of the country. However, some districts within the middle belt and southern belt continue to report high incidence of cases each year.

Female schistosomiasis cases continue to spatially move from the east-southern part of the country to the west-southern part of the country, with districts in the Savannah region decreasing in disease prevalence, while districts in the Bono East region consistently reported high burden of FS from 2020 to 2024. However, three districts in the northern region reported a yearly increase in FS burden from 2020 to 2023 before declining in 2024 (See Fig. 4).

### Assessing spatial autocorrelation of female schistosomiasis

Table 1 also shows a high level of clustering in the area (0.060032, Z-Score = 2.650757 and a *P* < 0.008). Furthermore, using spatial autocorrelation analysis and a z-score of 2.650757, this clustering pattern of female schistosomiasis cases is unlikely to be a coincidence (See Table 1). In assessing Local Indicators of Spatial Association (LISA), 499 number of permutations, and a distance band of 56,234.94 m were used. Clustering analysis using LISA showed that 10 districts with high prevalence surrounded by districts with a high prevalence of female schistosomiasis (high-high), and 4 and 9 districts showed high-low and low–high outlier clustering respectively. However, 18 districts were reported as having low-low clustering of female schistosomiasis (See Fig. 5).

**Table 1:** Spatial Autocorrelation Report Produced by ArcGIS pro with additional Global Moran’s Index Statistics, Ghana, 2020–2024

Moran’s Index	z-score	*P*-value	Outcome
0.060032	2.650757	0.008031*	Clustered

^*^Statistically significant

### Hotspot analysis

**Fig. 6:**
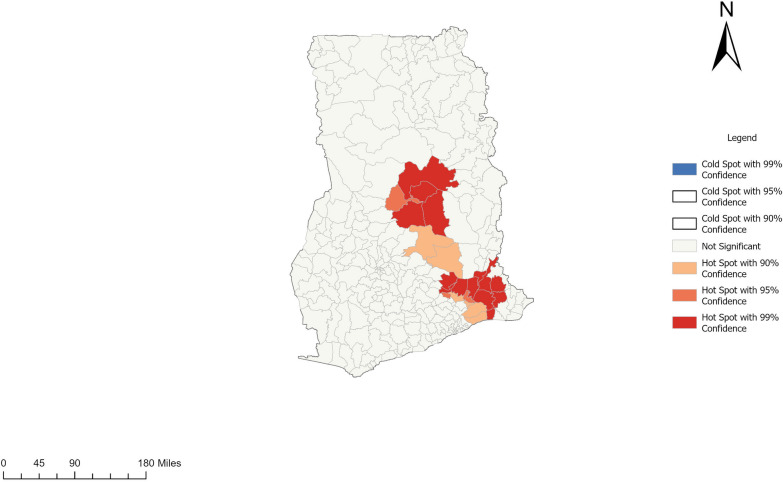
Getis-Ord Gi* cluster map showing hotspot for female schistosomiasis in districts in Ghana, 2020–2024

The Getis Ord Gi* analysis spatially estimates the concentration of female schistosomiasis cases. Figure 6 shows districts with statistically significant clusters (*P*-value < 0.005). Based on 95% and 99% confidence, statistical significance was determined. The stronger the grouping of low values, the lower the Z-score (cold spot). However, the more concentrated the grouping of high values, the higher the Z-score (hot spot).

Seventeen districts (*P*-value < 0.05) were identified as displayed in Fig. 6 representing red and deep pink clusters in the study area. The white portion in Fig. 5. shows areas that were not statistically significant. The map clearly shows two distinct clusters of hotspots within the study area. The first hotspot zone lies in the middle zone of the country, covering East Gonja municipality in the Savannah region, to Pru West, Pru East, Sene West, and Atebubu Amantin municipality in the Bono East region. At the same time, the second zone can also be seen with the south-western part of the country affecting Upper and Lower Manya Krobo districts, Fanteakwa North and South districts, Asuogyaman and Abuakwa North in the Eastern region; South Dayi, Adaklu, North and Central Tongu districts, and Ho West in the Volta region; and Ada West in the Greater Accra region (See Fig. 6 for details).

## Discussion

The present study revealed that there was significant inter-district variation of female schistosomiasis, with the annual parasite index (API) varying between 70.9 in 2022 per 10,000 to 99.0 per 10,000 in 2024 (See Fig. 2). This finding was higher than a study conducted in Volta region, Ghana [10] but lower than the findings of a study conducted among endemic countries in Africa [21]. This significant increase in case prevalence may be associated with limited access to praziquantel among the general population. Majority of mass drug administration (MDA) of praziquantel programmes mostly focuses on school children to the neglect of the general population who may have equal risk of being infected with *Schistosoma* [22]. Thus, infected persons serve as a source of infection for treated school children.

The current study also showed that the majority of FS cases were reported in the second half of the year. This phenomenon may be due to limited rainfall during such period, which leads people towade into water bodies at farther distances for clean water [16].

Additionally, majority of hotspot districts in the middle zone of the country cluster around the Volta Lake which may significantly serve as a source of infection for the population [10]. The Volta Lake serves as a water source for both household and economic activities. Traditionally, females are considered responsible for household chores. They are therefore responsible for fetching water from the Volta Lake [23]. This thus increases their risk of continuous point-source infection with *Schistosoma* parasitic worms.

The present study also revealed a significant trend in the epidemiology of female schistosomiasis, with a notable decline in cases in various districts of northern regions. The observed decline in female schistosomiasis cases in the northern part of the country may be attributed to a combination of absence of numerous water bodies in the northern part of the country and improved public health interventions [15, 24]. Coupled with a good community risk communication, improved coverage of praziquantel MDA for both community members and school children at school may significantly break the chain of transmission for schistosomiasis [15]. This may account for the observed declining trend in the prevalence of FS in the northern districts in Ghana. Contrarily, the middle and southern belts of the country continue to record higher prevalence of FS. Despite this observed difference, the disease prevalence of FS continues to largely move spatially from the east-southern to the west-southern part of the country. This may be attributed to increased agricultural activities coupled with persistent exposure to rivers and streams in these areas. Thus, entrenching a common continuous source infection with *Schistosoma* [25, 26]. It is therefore critical to prioritize culturally sensitive socio-environmental interventions that reduce exposures and improve treatment of infected persons in the west-southern part of the country.

In exploring statistically significant hotspots, 14 hotspots at 99% confidence level were discovered based on the aggregated temporal prevalence (2020–2024), whereas three at 95% confidence level were observed. This phenomenon illustrates that these districts, though having a high prevalence of female schistosomiasis, are also surrounded by districts with similar prevalence. Consequently, they may expect to exhibit this trend of high concentration of cases over time.

Hence, these hotspot zones are more likely to record local FS outbreaks if specific interventions are not employed to prevent future epidemics. However, a greater portion of the country did not have significant hotspots. This trajectory is expected since the cases were not evenly distributed in space. This finding should be a pointer for the health system to focus interventions and resources on the identified hotspots while maintaining surveillance across all districts.

Interestingly, Ashanti and Western region, that have high population density coupled with high number of water bodies recorded no significant hotspot of female schistosomiasis. High levels of illegal mining activities that deplete these reservoirs for *Schistosoma* and its intermediate host (water snail). Coupled with higher urbanization and improved social and agricultural activities, as well as higher literacy and improved water source in these regions compared to Bono East and Savannah where the majority of the population is rural, this can explain the phenomenon[27]. Second, most of the districts that recorded local-level clusters (*P* < 0.01 and 0.05) were found in the southern part of the country and thus proximate to tropical environmental conditions that expose them to increased contact with water bodies that may be infested with *Schistosoma*. These areas are more likely to have a higher number of rivers infested with copepods and *Schistosoma,* thus increasing environmental conditions necessary for *Schistosoma* transmission [14, 28]. For example, other studies have discovered that climatic conditions and differentiation among different districts were responsible for differing levels of schistosomiasis prevalence [13, 29]. While an attempt to establish natural limited exposures may be difficult, the study findings suggest an equitable distribution of resources, public education, mass drug administration, and improved healthcare facilities as the most feasible approach in thinning out the entrenched endemicity and its effect on the country as suggested by other researchers [24].

The districts with low prevalence are not entirely safe as time progresses, especially if higher prevalence districts surround them. This trend is of significant worry since they are likely to be impacted by the surrounding district, as it is evident that population movement introduces the disease to new geographical areas [3]. Regular surveillance is therefore needed to prevent mobility-based transmission [21]*.* Spatial outliers were examined using local-based clustering analysis (Anselin Local Moran’s *I*).

The current study showed that 10 districts in Bono East, Eastern, Volta, and Greater Accra had a high prevalence and were equally surrounded by high prevalence districts. This implies that these areas will continue to experience higher levels of endemicity if robust interventions are not implemented. More vigorous attention must therefore be accorded to these areas. It was also discovered that six districts had lower prevalence and were bordered by high-prevalence districts. Thus, urgent logistics allocation is needed to safeguard these low-prevalence districts. Four districts were reported to have high prevalence and surrounded by low-prevalence districts. Improved mass drug administration strategies, socio-environmental, and infrastructure to curb the possible transmission of cases to low-prevalence districts are key to setting FS endemicity anticlockwise. In most parts of the country, low-low clusters were observed. From the mid-north to the deep south, no significant outlier was observed regarding FS prevalence. Any policy hoping to achieve maximum output should target districts with spatial hotspots and outliers.

The study acknowledged some limitations. One significant limitation of the current study is the scale of data used (spatial resolution). Since we considered district-level centroids, there is a higher propensity to mask local-level, meaning no matter how hard we tried to optimize the modeling process. Also, ecological fallacy could not be prevented entirely. However, considering the scale district, the extent of such ecological fallacy is minimal. Again, the study primarily relied on DHIMS 2 data for FS cases. These cases may not accurately represent the disease burden at the community level. The population distribution was based on a projection from the 2010 (for 2020 and 2021) and 2021 (for 2022, 2023, and 2024) population and housing census, which may not also represent the actual population of the various units of analysis. Even better, the five-year trend of 4864 cases of female schistosomiasis were fit to conduct the analyses and generate the disease risk maps.

## Conclusions

The present study has strongly showed the uneven distribution of schistosomiasis infections among women in Ghana, pointing out the critical need to implement gender-sensitive interventions that address this high disease burden. Hotspots and temporal trend show continuous transmission of the disease despite implementation of MDA, suggesting that though current interventions are important, it may not be addressing localized transmission patterns and vulnerable population needs, such as women and girls. Thus, the critical need to visualize the burden and transmission pattern of schistosomiasis through spatial epidemiology tools at the primary healthcare level where control strategies are implemented. This will significantly help in identifying vulnerable populations, and guide resource allocation and intervention strategies.

The present study therefore reaffirms the essential role of data-driven, spatially targeted, and gender-sensitive local interventions as cost-effective measures. Hence, not only catalyzing Ghana’s effort to eliminating the disease by 2030, but reducing the burden of the disease among women and girls.

## Data Availability

The dataset used and/or analysed during the current study is available from the corresponding author on reasonable request.

## Abbreviations

API: Annual parasite index
DHIMS: District health information management system
FDR: False discovery rate
FGS: Female genital schistosomiasis
FID: Feature identifier
FS: Female schistosomiasis
GIS: Geographic information system
HIV: Human immunodeficiency virus
HPV: Human papillomavirus
IDSR: Integrated disease surveillance and response
LISA: Local Indicators of Spatial Association
M&E: Monitoring and evaluation
MDA: Mass drug administration
MMDAs: Metropolitan, Municipal, and District Assemblies
NTD: Neglected tropical disease
OID: Object identifier
SAC: School aged children
USA: United States of America
WHO: World Health Organization
